# The role of autonomy and social support in the relation between psychosocial safety climate and stress in health care workers

**DOI:** 10.1186/s12889-017-4484-4

**Published:** 2017-06-08

**Authors:** Bo M. Havermans, Cécile R. L. Boot, Irene L. D. Houtman, Evelien P. M. Brouwers, Johannes R. Anema, Allard J. van der Beek

**Affiliations:** 1 Department of Public and Occupational Health, Amsterdam Public Health research institute, VU University Medical Center, PO box 7057, 1007 MB Amsterdam, The Netherlands; 2 Body@Work, Research Center Physical Activity, Work and Health, TNO-VU University Medical Center, Amsterdam, The Netherlands; 3 Netherlands Organisation for Applied Scientific Research, TNO, Leiden, The Netherlands; 4 School of Social and Behavioural Sciences, Tranzo, Tilburg University, Tilburg, The Netherlands

**Keywords:** Psychosocial safety climate, Social support, Autonomy, Stress, Employee, Health care

## Abstract

**Background:**

Health care workers are exposed to psychosocial work factors. Autonomy and social support are psychosocial work factors that are related to stress, and are argued to largely result from the psychosocial safety climate within organisations. This study aimed to assess to what extent the relation between psychosocial safety climate and stress in health care workers can be explained by autonomy and social support.

**Methods:**

In a cross-sectional study, psychosocial safety climate, stress, autonomy, co-worker support, and supervisor support were assessed using questionnaires, in a sample of health care workers (*N* = 277). Linear mixed models analyses were performed to assess to what extent social support and autonomy explained the relation between psychosocial safety climate and stress.

**Results:**

A lower psychosocial safety climate score was associated with significantly higher stress (B = −0.21, 95% CI = −0.27 – -0.14). Neither co-worker support, supervisor support, nor autonomy explained the relation between psychosocial safety climate and stress. Taken together, autonomy and both social support measures diminished the relation between psychosocial safety climate and stress by 12% (full model: B = −0.18, 95% CI = −0.25 – -0.11).

**Conclusions:**

Autonomy and social support together seemed to bring about a small decrease in the relation between psychosocial safety climate and stress in health care workers. Future research should discern whether other psychosocial work factors explain a larger portion of this relation.

**Trial registration:**

This study was registered in the Netherlands National Trial Register, trial code: NTR5527.

## Background

Health care workers are exposed to psychosocial work factors, such as workload and social support [1, 2], which are associated with stress [3–5]. Stress is a risk factor for adverse physical and mental health effects [6–8]. Stress contributes substantially to sickness absence [9], and poses a financial threat to organisations and society at large, due to related productivity loss and health care costs [10]. Psychosocial work factors have been defined as social characteristics of the work environment that interact with individual, psychological factors [11]. Influential work stress models, such as the Job Demand-Control (−Support) Model [12], and the Job Demands-Resources model [4], show through which mechanisms psychosocial work factors can influence stress in employees.

In recent studies, it is argued that psychosocial work factors are a consequence of an aspect of the psychosocial work environment, called the Psychosocial Safety Climate (PSC) [13], which is defined as the readiness of management to prevent and respond to stressful working conditions [14]. Studies have revealed that PSC is a construct that is distinct from psychological and physical safety, as well as organisational support [15], and that PSC is associated with psychosocial work factors, such as job demands [13], job control, and supervisor support [16, 17]. In turn, low PSC has been found to be predictive of psychological distress and burnout [16, 18]. Although these studies provide interesting insights, the understanding of how PSC might affect stress in workers needs to be expanded. Studying the relation between PSC and stress is vital to this understanding.

As PSC is argued to exert its influence on stress-related mental health outcomes through psychosocial work factors [16], understanding the relation between PSC and stress requires that psychosocial work factors are taken into account in this relation. Because the psychosocial work environment is not limited to one level, it could be useful to incorporate psychosocial work factors that represent different levels of the psychosocial work environment, such as the level of the task, the employee level, and the organisational level (such as management).

The three levels mentioned above (i.e. task, employee, organisation) are represented by the psychosocial work factors autonomy and social support by colleagues and supervisor [4, 12]. Autonomy represents the task level, and has been found to be negatively associated with stress in employees [19–21]. In the literature, a distinction has been made between co-worker support (employee level) and supervisor support (organisational level) [22]. Nieuwenhuijsen et al. [23] found that low co-worker support and low supervisor support predicted the incidence of stress-related disorders. Other studies have demonstrated support to be negatively associated with work stress, and positively associated with job satisfaction [24–26]. Taking into account these two types of support and autonomy can result in improved understanding of the relation between PSC and stress. The current study assesses to what extent the relation between PSC and stress in health care workers can be explained by autonomy and social support.

## Methods

### Study design, participants, and procedure

This cross-sectional study used baseline data from a controlled trial of Stress Prevention@Work. Stress Prevention@Work is a project aimed at developing and implementing a multi-faceted, integral implementation strategy for organisational work stress prevention. The trial started in May 2016, and was carried out within a large (>4500 employees) Dutch health care institution. Participants were health care personnel, working in care teams at various locations throughout the Netherlands. They were eligible for participation in the current study if: 1) they were part of one of the teams that participated in the trial, and 2) had no missing values for the scales used in this study. Participants were approached by email to fill out an online questionnaire, which took approximately 16 min to complete. A maximum of two reminders was sent by email. Some participants indicated that they would rather fill out a paper version of the questionnaire, which was subsequently provided to them. Incentives provided for participation were cakes for teams that reached a minimum response of 70%. Of 473 employees that were approached, 304 participants filled out the questionnaire (response rate: 64%). A small number of respondents (*n* = 31) filled out the paper version of the questionnaire. Participants who did not complete the questionnaire (*n* = 27) were omitted from the analyses because of missing values, leaving 277 participants as the final study sample. The trial was approved by the Medical Ethical Committee of the VU University Medical Center, Amsterdam, the Netherlands.

### Stress

The primary outcome of the current study was stress, which was measured using the seven-item stress sub-scale of the short version of the Depression Anxiety and Stress Scale (DASS-21)(items can be found in [27], p. 340). The DASS-21 has been validated in non-clinical settings [28, 29]. The stress sub-scale of the DASS-21 has seven items assessing the stress experienced in the past week. The items are statements, such as “I found it hard to wind down”, and “I found myself getting agitated”. Participants indicated how much the statement applied to them on a Likert-scale that ranged from 0 (“Never”) to 3 (“Almost always”). This resulted in a scale score that ranged from 0 to 21, with a higher score representing more stress.

### Psychosocial safety climate (PSC)

The PSC-12 [30] was used to measure PSC. This is a 12-item questionnaire that assesses PSC by addressing four factors: (1) management support for stress prevention (“Senior management show support for stress prevention through involvement and commitment”); (2) health and safety over production goals prioritisation by management (“Psychological well-being of staff is a priority for this organization”); (3) organisation’s interest for employee contributions (“Information about workplace psychological well-being is always brought to my attention by my manager/supervisor”); and (4) participation and involvement of the organisation (“In my organization, the prevention of stress involves all levels of the organization”) (items can be found in [30], p. 363). A Dutch translation of the PSC-12 was obtained from another research team who collaborated with the original developer in the translation process. Together with that team, and with the original developer of the scale, the Dutch version of the PSC-12 was fine-tuned for use in the present study. The PSC-12 was adapted to represent the organisational structure more accurately (i.e. “supervisor” was replaced by “team coach”). Participants indicated how much they agreed with the statements on a Likert-scale that ranged from 1 (“strongly disagree”) to 5 (“strongly agree”). The scale score of the PSC-12 is the sum of all 12 items, ranging from 12 to 60. The Dutch version of the scale had high reliability (Cronbach’s Alpha = .91).

### Social support and autonomy

Social support was measured using two sub-scales from the Dutch version of Job Content Questionnaire (JCQ) [22] (items can be found in [31]). The first sub-scale, measuring co-worker support, contained four statements (e.g. “People I work with are helpful in getting the job done”). The second sub-scale, measuring supervisor support, also contained four statements (e.g. “My supervisor pays attention to what I am saying”). Autonomy was also measured using a subscale from the JCQ [22]. It contained three statements (e.g. “My job allows me to make a lot of decisions on my own”). For all sub-scales, participants indicated how much they agreed with the statements on a Likert-scale that ranged from 1 (“strongly disagree”) to 4 (“strongly agree”). For both support sub-scales, scale scores were the sum of the four items, and both ranged from 4 to 16. The scale score of the autonomy sub-scale was the sum of the three items, with a range of 3 to 12.

### Potential confounders

Common individual factors [32] were assessed as potential confounders. These factors were age (measured as acontinuous variable), gender (male/female), and education. Education was categorised into three categories: low (i.e. lower general secondary education, preparatory secondary vocational education), moderate (i.e. intermediate vocational training, higher general secondary education, pre-university education), and high (i.e. higher vocational education, university education) [33].

### Analyses

Descriptive statistics were used to report on the study sample’s age, gender, education, PSC, co-worker support, supervisor support, autonomy, and stress. In addition, Pearson Correlations between stress, PSC, co-worker support, supervisor support, and autonomy were calculated, and collinearity diagnostics (Variance Inflation Factor, or VIF, was calculated for all scales, taking into account each scale as a dependent variable) were performed to check for multicollinearity. Generally, a VIF between 5 and 10 is considered problematic for the interpretation of the results [34]. Linear mixed models analysis was performed to assess the univariable association between PSC and stress (Model 1). Then, gender, age and education were separately added to the model to check for confounding. If the regression coefficient of PSC and stress changed more than 10% by adding any of these possible confounders, then the confounder was added to the model. Interactions between PSC and age, and between PSC and education were tested to check for effect modification. If interaction terms were statistically significant (*p* < 0.05), effect modification was assumed, and subgroup analyses were performed. Because the primary outcome (i.e. stress) was not normally distributed (right-skewed), bootstrapped analyses were performed (bias corrected and accelerated; 5000 samples) to check the robustness of the models [35].

To assess if a substantial proportion of the association between PSC and stress could be explained by co-worker support, co-worker support was added to the model (Model 2). If the regression coefficient of PSC and stress changed more than 10%, it was assumed that co-worker support changed a substantial part of the association between PSC and stress. The same procedure was followed for supervisor support (Model 3) and autonomy (Model 4). In the final two models, co-worker and supervisor support were both added (Model 5), and autonomy was added as well (Model 6). A random intercept at team level was included in all six models, to control for team differences in PSC. If the model with the intercept showed significant improvement compared to the model without the random intercept (i.e. a significant reduction of the −2 Log Likelihood value), the model with the random intercept was reported. All analyses were performed using IBM SPSS 22.

## Results

The study sample included 267 (96%) women, and the average age was 43.3 (SD = 11.2) years. The majority of the participants (90%) had moderate education, while 26 participants (9%) had high education. Table 1 shows descriptive results for PSC, stress, co-worker support, supervisor support, and autonomy.

**Table 1:** Mean, standard deviation and range of psychosocial safety climate, stress, supervisor support, co-worker support, and autonomy (*n* = 277)

	Mean	Standard Deviation	Range (min – max)
Psychosocial Safety Climate	30.95	7.47	12 – 52
Stress	4.19	4.14	0 – 21
Co-worker Support	12.34	1.75	4 – 16
Supervisor Support	10.61	2.12	7 – 16
Autonomy	8.38	1.32	4 – 12

### The relation between PSC and stress, social support, and autonomy

The correlations between stress, PSC, co-worker support, supervisor support, and autonomy are presented in Table 2. The VIF did not exceed 1.489, indicating that there was no multicollinearity.

**Table 2:** Correlations between PSC, co-Worker support, supervisor support, autonomy, and stress

	PSC^a^	Co-Worker Support	Supervisor Support	Autonomy
Stress	−.37	−.07	−.23	−.22
PSC^a^	1	.09	.51	.28
Co-Worker Support	-	1	.09	.15
Supervisor Support	-	-	1	.37

^a^PSC = Psychosocial Safety Climate

The six models of the association between PSC and stress are presented in Table 3. There was no confounding or effect modification by gender, age and education. Therefore, adjustment of the models was not necessary. Moreover, adding a random intercept for PSC at team level did not significantly improve any of the six models. Therefore, the models without random intercepts are presented here. Higher PSC was associated with a significantly lower stress score in the univariable model (Model 1; B = −0.21, 95% CI = −0.27 – -0.14). Adding co-worker support to the model did not result in a change larger than 10% in the B coefficient of PSC and stress (Model 2; B = −0.20, 95% CI = −0.26 – -0.14). This was also the case for supervisor support (Model 3; B = −0.19, 95% CI = −0.26 – -0.12), for autonomy (Model 4; B = −0.19, 95% CI = −0.25 – -0.12), and for the model which included both supervisor and co-worker support (Model 5; B = −0.19, 95% CI = −0.26 – -0.12). The full model, which included social support and autonomy (Model 6; B = −0.18, 95% CI = −0.25 – -0.11), showed a 12% decrease in the B coefficient of PSC and stress, compared to that same coefficient in Model 1 that did not contain support and autonomy. All models were robust for the non-normal distribution of stress.

**Table 3:** Autonomy, co-worker and supervisor support in the relation between psychosocial safety climate and stress

	Model 1		Model 2		Model 3		Model 4		Model 5		Model 6	
	B	95%-CI	B	95%-CI	B	95%-CI	B	95%-CI	B	95%-CI	B	95%-CI
PSC^a^	−.21*	−.27 – -.14	−.20*	−.26 – -.14	−.19*	−.26 – -.12	−.19*	−.25 – -.12	−.19*	−.26 – -.12	−.18*	−.25 – -.11
*Co-worker Support*			−.08	−.35 – .18					−.08	−.34 – .19	−.04	−.31 – .22
*Supervisor Support*					−.11	−.36 – .14			−.10	−.36 – .15	−.03	−.29 – .23
*Autonomy*							−.41**	−.77 – -.05			−.39	−.77 – .02

^a^ *PSC* Psychosocial Safety Climate

*p < 0.001; ** *p* < 0.05

## Discussion

The aim of this study was to assess to what extent the relation between PSC and stress in health care workers can be explained by autonomy and social support. A lower PSC score was significantly associated with higher stress in health care workers. Neither co-worker support, supervisor support, nor autonomy explained this association. Taken together, however, social support and autonomy diminished the relation between PSC and stress by 12%.

Our study population was comparable to other samples of health care workers with regard to levels of stress and psychosocial work factors. The stress score found in the current study was comparable to that of another sample of health care workers [36], as well as to that of a large, more general sample [28]. Comparable scores were also found for supervisor support and co-worker support in other studies of health care personnel [37–39]. The level of autonomy appeared slightly lower (almost 10%) compared to another study on health care workers [40]. The PSC score reported in the current study was also somewhat lower than those of other study samples from the health care sector (ranging between 9 and 18% lower) [15, 16], and from other sectors (ranging between 22 and 28% lower) [13, 17]. This might be due to an organisational change that had recently taken place. Following a restructuring in January 2016, a new managerial structure was introduced, that put the day-to-day management responsibility on teams themselves. By removing a layer of management (i.e. the direct supervisors) from the organisation, the distance between employees and management may have been increased. It is possible that different associations between PSC, stress, autonomy, and social support would be found in a sample with a high PSC score.

The finding of the current study, that supervisor support did not change the relation between PSC and stress, corresponds with previous research. Dollard and colleagues [16] found that supervisor support did not mediate the relation between PSC and psychological distress. Interestingly, social support and autonomy did diminish the strength of the relation between PSC and stress by 12%, when they were taken together. Perhaps, autonomy and social support explain unique portions of this relation.

Other psychosocial work factors than autonomy and social support may play a more defining role in the relation between PSC and stress. These might be other job resources, such as participation and rewards [4], or it might be job demands. Idris et al. [15] reported that job demands mediated the effect of PSC on psychological health problems. Dollard et al. [16] found that emotional demands and workload mediated the relation between PSC and emotional exhaustion. These studies suggest that demands could be more relevant to the relation between PSC and stress than resources. Alternatively, a combination between resources and demands may be necessary to explain the relation between PSC and stress.

Another way in which psychosocial work factors can be distinguished from one another, is the level of the psychosocial work environment they represent. This study included psychosocial work factors at the level of the task, the employee, and the organisation. Even though it could not be determined if any of these levels is more relevant to the relation between PSC and stress based on the current findings, it is possible that this determination can be made using other psychosocial work factors. Taking into account different levels, and also the balance between resources and demands, can provide a more specific insight into the mechanisms through which PSC may affect stress.

### Strengths and limitations

To the authors’ best knowledge, this is the first study to report on the PSC-12 translated into Dutch, also providing the first scores from Dutch employees. This study assessed the association between PSC and stress using the stress sub-scale of the DASS-21. By doing so, it strengthened the convergent validity of the association between PSC and stress, as this was the first time the DASS-21 was used to study this association.

Due to its cross-sectional design, no causal inferences could be made in the current study. Because non-respondents may not have participated due to high workload or stress, it is possible that stress in our sample was underestimated. The study sample was quite homogeneous with respect to gender (women) and educational level (moderate). This could limit the generalisability of the findings. However, the average stress score found in this study resembled the stress score of a non-clinical sample, which contained almost as much men (48%) as women [28]. Therefore, it is unlikely that study sample homogeneity, with respect to gender and educational level, has substantially impacted the findings. Still, caution is advised in interpreting and generalising the findings, until replication using more heterogeneous groups (for instance, with respect to gender, educational level, and job status), is realised. Moreover, as variability was greatest in stress and PSC, the likelihood of finding a primary effect was greater than finding a change in the relationship between PSC and stress by adding (the less variable) social support and autonomy. Finally, we cannot fully exclude that the study results might be biased by a coinciding restructuring of the managerial structure to self-guiding teams.

### Implications

This study adds to the understanding of PSC and stress, confirming their association. Future studies could assess whether psychosocial work factors other than social support and autonomy, such as psychological job demands, emotional demands, and organisational justice, or demands and resources combined, are more suitable for giving insight into the way PSC affects stress in employees. Additionally, the results of this cross-sectional study need replication using a longitudinal design.

The fact that, taken together, social support and autonomy did reduce the relation between PSC and stress has some value for practice. We hypothesize that autonomy or social support alone are not sufficient to reduce the association between PSC and stress. Our study results point in the direction that supportive psychosocial work factors at multiple levels are needed to counteract negative effects of PSC on stress. Therefore, stress prevention practice could benefit from a more complete insight into which psychosocial work factors are relevant to the association between PSC and stress.

## Conclusions

Lower PSC scores were associated with more stress in health care workers. Social support and autonomy seemed to bring about just a small decrease in the relation between PSC and stress. Future research should discern whether other psychosocial work factors explain a larger portion of this relation.

## Abbreviations

95% CI: Confidence Interval (95%)
B: Beta
DASS-21: Depression Anxiety and Stress Scale (21-item version)
JCQ: Job Content Questionnaire
PSC: Psychosocial Safety Climate
PSC-12: Psychosocial Safety Climate scale (12-item version)
SD: Standard Deviation
